# Epigenetic Alteration and its Association With Downregulated FOXP3 Gene in Indian Breast Cancer Patients

**DOI:** 10.3389/fgene.2021.781400

**Published:** 2021-11-29

**Authors:** Sadaf, Naseem Akhter, Raed A. Alharbi, Abdulmajeed A. A. Sindi, Mohammad Zeeshan Najm, Fahad A. Alhumaydhi, Mohammad Aasif Khan, S.V.S Deo, Syed Akhtar Husain

**Affiliations:** ^1^ Department of Biosciences, Jamia Millia Islamia, New Delhi, India; ^2^ Department of Laboratory Medicine, Faculty of Applied Medical Sciences, Albaha University, Albaha, Saudi Arabia; ^3^ School of Biosciences, Apeejay Stya University, Gurugram, India; ^4^ Department of Medical Laboratories, College of Applied Medical Sciences, Qassim University, Buraydah, Saudi Arabia; ^5^ Department of Surgical Oncology, All India Institute of Medical Sciences, New Delhi, India

**Keywords:** PCR-real time, methylation specific PCR (MS PCR), immunohistochemistry (IHC), biomarkers, mammary cancer, gene experession

## Abstract

**Background:** *FOXP3* gene, known to be a potential tumor suppressor, has been identified to interact with HER2 in mammary cancer. Moreover, the high expression of *FOXP3* serves as a good predictor of the survival of patients in breast cancer, prostate cancer, and gastric cancer. The expression and epigenetic alterations were evaluated in female breast cancer patients.

**Material and Methods:** Expression studies at the mRNA level and protein level were conducted in 140 breast cancer cases by real-time PCR and immunohistochemistry, respectively. Epigenetic studies were also conducted by analyzing the methylation status at the promoter region of the gene using MS-PCR.

**Results:** *FOXP3* mRNA expression and protein expression were downregulated in breast cancer patients. The absence of *FOXP3* protein expression is significantly associated with promoter methylation, where 70 methylated cases exhibited protein loss (70/95, 73.6%). Statistically, we also found a significant correlation between *FOXP3* protein expression and TNM stage, promoter methylation, and histological grade. The methylated *FOXP3* cases that did not express protein were also significantly associated with positive lymph node metastasis and HER-2 status.

**Conclusion:** The expression profile of *FOXP3* may serve as a prognostic factor. In short, *FOXP3* may stand in the most crucial list of biomarkers for breast cancer, bringing compelling results in terms of treatment and management of the disease.

## Introduction

It is a well-known fact that females around the world are mostly affected by breast cancer (1.7 million cases, 11.9%); however, it positions fifth as the cause of death (6.4%) because of the comparatively conducive prognosis. Whereas breast cancer accounted for the most prominent cause of mortality in females in undeveloped and developing regions of the world (Ferlay et al., 2015), the development of biomarkers and their clinical use in therapy for prediction and expected response hold a remarkable potential.

*FOXP3*(Forkhead box P3), located on Xp11.23, is a member of the Forkhead/winged-helix family of transcription factors which is responsible for X-linked autoimmune diseases in mice as well as humans (Wildin et al., 2001; Brunkow et al., 2001; Schubert et al., 2001). Transcription factor *FOXP3* regulates the development and function of Treg cells, and Treg cells are known to regulate homeostasis (Chen et al., 2015) and immunosuppression (Müller et al., 2010) and also recognized as the most peculiar marker for Treg (Hori et al., 2003). Before the advanced research on *FOXP3* expression, it was thought to be expressed only in hematopoietic cells but now seems to be present in human tumors, particularly tumors of the breast (Merlo et al., 2009). In mammary cancers, *FOXP3* is found to regulate *HER-2* and *SKP2* by repressing their expression, and importantly these genes are linked to a poor prognosis in the cases with breast carcinoma (Martin et al., 2010). The downregulation and many functional somatic mutations in the *FOXP3* gene were usually found in human breast cancer samples. These mutations may also account for the overall down-regulation at the protein level (Karanikas et al., 2008).

*FOXP3* halts the transcription of *HER-2* by attaching to the promoter region of the *ERBB2* gene (Redpath et al., 2011; Zuo et al., 2007), and it is a well-known fact that *HER-2* is a potent marker in terms of prediction and effective therapy (Presson et al., 2011; Weigelt and Reis-Filho, 2010). The study has also pointed out the high expression of *FOXP3* as a good predictor of the survival of a patient in prostate cancer, breast cancer, gastric cancer, and bladder cancer (Li et al., 2007; Wang et al., 2009; Winerdal et al., 2011; He et al., 2013; Fiori Lopes et al., 2014; Hao et al., 2014; Ma et al., 2014). Previous studies also reported that many SNPs in the *FOXP3* gene had been associated with breast cancer (Jiang and Ruan, 2014).

*FOXP3* is also linked to *p21* and *LATS2*, where it is involved in transcriptional control (Li et al., 2011). Due to these captivating characteristics of *FOXP3*, the present work examines the correlation of *FOXP3* protein expression with the clinicopathological variables to strengthen its role as a putative biomarker for breast carcinoma in the Indian population.

## Methodology

### Ethical Statement

The University Ethical Committee of Jamia Millia Islamia (JMI), New Delhi, and the Ethical Committee for Human Study of AIIMS (All India Institute of Medical Sciences), New Delhi, have officially approved the study. The experimental work had been undertaken with written consent obtained from each subject, and the study complies with the rules and standards set by the Ethics Code of the Medical Association of the world, which have been noted as per the Declaration of Helsinki as published in British Medical Journal (1964).

### Sample Collection

A total of 140 participants were included in the present case–control study. Cancer tissue from the breast and non-cancerous adjacent tissue were both obtained from the surgical oncology department of the collaborating institute (AIIMS). The samples were collected in three vials containing RNALater, phosphate-buffered saline (PBS), and formalin, respectively, for further processing.

The classification of breast cancer stages was done under the TNM staging system, and the histological grading of tumors was classified based on the Nottingham grade system. The exclusion criteria in the current study included familial cancer, any previous type of cancer, other metastasized cancer that has spread from different organs, and chemotherapy and radiotherapy exposure. In addition, included were various clinicopathological variables such as tumor distinctiveness [age, tumor size, metastasis at the lymph node level, TNM staging, grade of tumor, molecular subtype of tumor, hormonal receptor status (ER, PR, and Her2neu), and reproductive history (menopausal status parity)].

### Quantitative- PCR

RNALater (Qiagen) was used to store excised tissues from normal and breast cancer patients, and then RNA was extracted by using the TRIzol method as per the manual. cDNA was synthesized using a Thermo Fisher verso kit from the extracted RNA. The qPCR is processed using Roche LightCycler® 96 machine with SYBR Green I Master mix reagent (Roche) with the help of *FOXP3* primers (Fwd- 5′-TCC​CAG​AGT​TCC​TCC​ACA​AC-3′ and Rev-5′ATTGAGTGTCCGCTGCTTCT-3′) that give an amplified 122-bp product. The internal control used was GAPDH gene which was also amplified in the same PCR reactions. The program used for the amplification cycles was as follows: preheating at 95°C for 1 min, 30 cycles of denaturation at 95°C for 20 s, annealing at 58°C for 15 s, extension at 72°C for 15 s, and further elongation at 72°C for 7 min. The experiments were repeated thrice.

The relative quantification of expression was calculated as the calibrator normalized ratio using LightCycler 96 (Roche) Software 1.5. The formula used, RQ = 2^–∆∆C^t, was according to MIQE.

### Genomic DNA Extraction

The phenol-chloroform extraction method was used to isolate high-molecular-weight total gDNA from both tumor and normal tissues stored in PBS. The genomic DNA isolated was quantified on a Nanodrop spectrophotometer, and its quality was also assessed using an A260/280 ratio. It was further visualized on the 1% agarose gel stained with ethidium bromide under a UV transilluminator.

### MS-PCR for Epigenetic Analysis

Isolated gDNA from the tissues were given bisulfite treatment using Zymo research EZ DNA Methylation-Gold™ Kit per the instructions. The treated gDNA was amplified using two sets of methylated and unmethylated primers for the FOXP3 promoter. MethPrimer tool was used to design the set of primers for methylation and unmethylation. (Li and Dahiya, 2002). The methylated primer pairs for the promoter region of the *FOXP3* gene were: forward 5′- TGT​AGG​GGG​TGT​AGA​ATT​TTT​TTC-3′ and reverse 5′- AAA​CTA​AAT​TCC​CAA​AAA​CCT​CG-3′ and for the unmethylated were forward 5′- GTA​GGG​GGT​GTA​GAA​TTT​TTT​TTG​T-3 and reverse 5′- TAA​AAC​TAA​ATT​CCC​AAA​AAC​CTC​A-3′. The positive controls used in the experiment were commercially available Completely methylated and unmethylated human genomic DNA.

MS-PCR was performed under the following cycles: First denaturation at 95°C for 7 min, denaturation at 95°C for 30s 52.5°C annealing for both types of primers for 30 s, 72°C for 30 s and final elongation at 72°C for 7 min which was amplified for 35 cycles. Then, 2% agarose gel stained with EtBr was used to visualize and analyze the PCR product, which was finally photographed using the Bio-Rad Gel Documentation system. All experiments were conducted in triplicates.

### Immunohistochemistry

The tissue biopsies of the breast carcinoma and adjoining non-cancerous tissue were conserved in formalin-fixed blocks. The blocks were then sectioned and engraved on slides coated with poly-L-lys and exposed to deparaffinization by dipping in different concentrations of xylene and further rehydrated with grades of ethanol. By using 0.3% H_2_O_2_ for 30 min, the endogenous peroxidase activity was blocked, and sodium citrate buffer (pH 9.0) was used for Ag retrieval.

The sections were treated for 30 min with TENG-T (10 mM Tris; 5 mM ethylenediaminetetraacetic acid, 0.15 mol/L NaCl, 0.25% gelatin, and 0.05% (v/v) Tween20 (pH 8.0) to block the samples. Bovine serum albumin was used to limit unspecific binding to the protein. The slides were treated with the primary antibody (mAbCam#ab22510 *FOXP3* 1:50) and incubated overnight at 4°C. The slides were then treated for 30 min with a biotinylated anti-mouse secondary antibody along with streptavidin–horseradish peroxidase conjugate. The DAB chromogen was finally used as a substrate to give a brown-colored precipitate. The slides were also treated with hematoxylin as a counterstain for better contrast.

Histopathologists interpreted the slides after immunohistochemistry; the slides were photographed under a light microscope at ×400 magnification. The pathologist further graded the expression on the number scale 0–4, with 0 as no expression and 4 as highest expression; the slides with >50% protein staining were considered in the highest scale.

### Statistics

SPSS version 22.0 for Windows was used for all the statistical correlations between the outcomes and the clinicopathological parameters. All data were expressed as mean ± standard error. Fisher’s exact test was used to obtain *p*-values between mRNA levels, methylation status, and protein expression with the clinicopathological parameters. Non-parametric Wilcoxon signed-rank test is used to estimate *FOXP3*/*GAPDH* mRNA expression levels significantly in both cancer and normal tissue samples. The *p*-values >0.05 were considered as significant.

## Results

### mRNA Expression of the FOXP3 Gene Is Downregulated in the Cases of Breast Cancer

**FIGURE 1:**
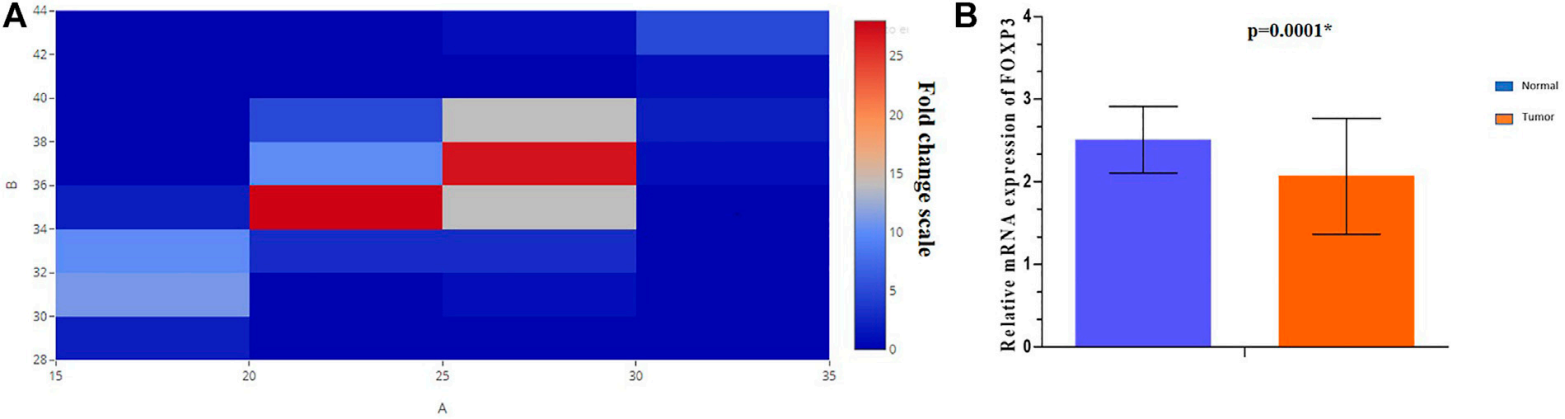
**(A)** Heat map plot showing the mRNA relative expression of the *FOXP3* gene (fold change) in breast cancer patients. **(B)** Graphical representation of the relative expression of *FOXP3*/*GAPDH* mRNA in breast tumor and adjoining non-tumorous tissues.

*FOXP3* mRNA expression revealed downregulation in 63.5% (89/140) of cases (Figure 1A), out of which nearly 86.5% (77/89) of the cases registered in the study were linked to histological grade type 1 and type 2. As per the fold change analysis, 89 cases out of 140 samples seemed to be downregulated (5.09-fold), while the expression pattern of *FOXP3* at the level of mRNA, when normalized accordingly with the internal control *GAPDH* in tumor and non-tumor tissues, was 2.07 ± 0.7 (mean ± standard error) and 2.51 ± 0.4 (mean ± standard error) (Figure 1B), (*p*-value of <0.0001; Table 1) respectively. However, there was no significant association observed between *FOXP3* mRNA level and various clinical variables.

**TABLE 1:** Correlation study of *FOXP3* mRNA expression with clinicopathological parameters in North Indian breast cancer patients.

Characteristics	Total (N)	*FOXP3* mRNA expression relative to GAPDH (mean ± S.E)	*p-*value
Tissue^a^			
Normal	89	2.51 ± 0.4	<0.0001*
Tumor	89	2.07 ± 0.7	
Age			
<50	50 (35.71)	2.40 ± 0.60	0.716
≥50	90 (64.29)	2.50 ± 0.40	
Menopausal status			
Premenopausal	40 (28.57)	3.07 ± 0.80	0.698
Postmenopausal	100 (71.43)	2.23 ± 0.40	
Estrogen receptor status			
Negative	37 (26.42)	2.79 ± 0.80	0.691
Positive	103 (73.58)	2.36 ± 0.42	
Progesterone receptor status			
Negative	67 (47.85)	2.36 ± 0.51	0.862
Positive	73 (52.15)	2.58 ± 0.58	
Her2 neu status			
Negative	70 (50)	2.65 ± 0.55	0.726
Positive	70 (50)	2.30 ± 0.55	
Tumor size			
<5	64 (45.71)	1.50 ± 0.35	0.482
≥5	76 (54.29)	3.29 ± 0.64	
Lymph node status			
Positive	119 (85)	2.68 ± 0.45	0.811
Negative	21 (15)	1.37 ± 0.40	
TNM staging			
Stage (I + II)	45 (32.1)	2.87 ± 0.73	0.192
Stage (III + IV)	95 (67.9)	2.28 ± 0.46	
Histological grade			
(I + II)	120 (85.7)	2.47 ± 0.42	0.803
(III)	20 (14.3)	2.51 ± 1.02	
Molecular subtypes			
Luminal A	51 (36.44)	2.13 ± 0.56	0.193
Luminal B	53 (37.86)	2.58 ± 0.64	
Her2neu enriched	18 (12.85)	1.48 ± 1.04	
TNBC	18 (12.85)	4.24 ± 1.44	

TNBC: triple negative breast cancer, FOXP3: Forkhead Box P3.

a Only downregulated cases were included.

### Expression of FOXP3 Protein is Either Lost or Low in Breast Cancer

**FIGURE 2:**
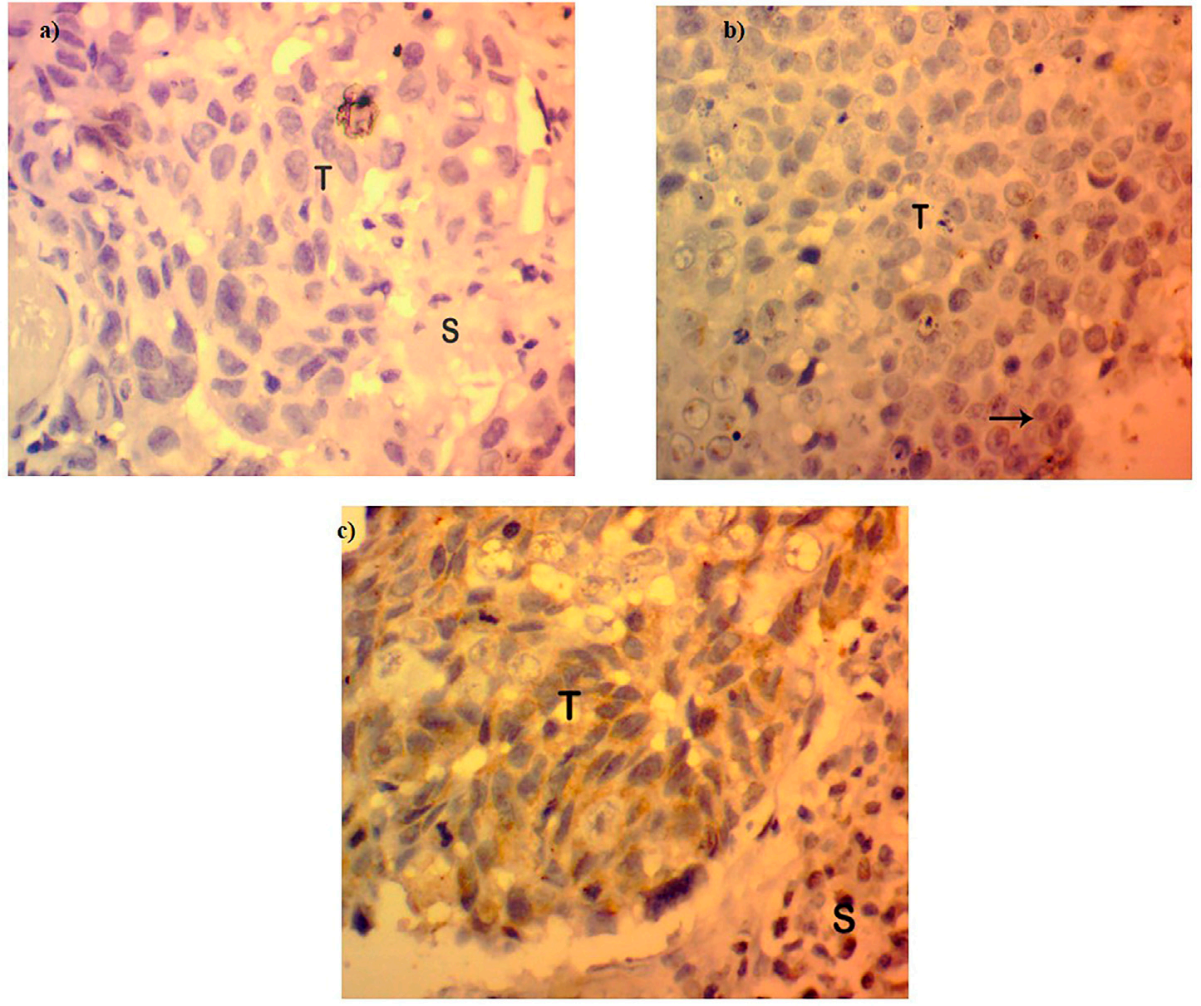
Protein expression in *FOXP3* by immunohistochemistry. **(A)** Absence or loss of *FOXP3* expression in Breast cancer tissues. **(B)** *FOXP3* low expression in breast tumor tissues. **(C)** *FOXP3* moderate/high expression in breast tumor tissues.

The expression of *FOXP3* at the protein level was found to be either low or absent in 95 cases of the total 140 samples involved in the study (67.85%) (Figure 2), while in the other 45 cases, the expression pattern was either in the high or moderate range as interpreted by a histopathologist on the basis expression scale (45/140, 32.14%). The protein expression pattern was in relation to the mRNA expression. The *FOXP3* protein, as visualized by immunohistochemistry, was mainly located in the nuclear region.

### FOXP3 Protein Expression and Its Significant Correlation With Clinicopathological Parameters

As revealed by immunohistochemistry staining, the majority of *FOXP3* proteins in the samples were found to be significantly downregulated. Moreover, when we tried to statistically associate the protein expression with the clinical parameters of the patients, we observed a significant correlation between TNM staging and *FOXP3* protein expression (*p* < 0.035) (Table 2). However, with other parameters, no significant association was obtained statistically (Table 2), though most cases with tumor grades 1 and 2 seem to have protein loss (82/95, 86.3%).

**TABLE 2:** Statistical analysis of the protein expression pattern of *FOXP3* with the clinicopathological variables of patients with breast cancer.

Characteristics	Total cases (N)	*FOXP3* absent	*FOXP3* present	*p*-value
Age				
<50	50	36 (72)	14 (28)	0.457
≥50	90	59 (65.5)	31 (34.5)	
Menopausal status				
Premenopausal	40	27 (67.5)	13 (32.5)	1.0
Postmenopausal	100	68 (68)	32 (32)	
Estrogen receptor status				
Negative	37	26 (70.2)	11 (29.8)	0.838
Positive	103	69 (66.9)	34 (33.1)	
Progesterone receptor status				
Negative	67	43 (64.2)	24 (35.8)	0.469
Positive	73	52 (71.2)	21 (28.8)	
Her2 neu status				
Negative	70	44 (62.8)	26 (37.2)	0.278
Positive	70	51 (72.8)	19 (27.2)	
Tumor size				
<5	64	46 (71.8)	18 (28.1)	0.37
≥5	76	49 (64.4)	27 (35.6)	
Lymph node status				
Positive	119	80 (67.2)	39 (32.8)	0.804
Negative	21	15 (71.5)	6 (28.5)	
TNM staging				
Stage (I + II)	45	25 (55.5)	20 (44.5)	0.035*
Stage (III + IV)	95	70 (73.6)	25 (26.4)	
Histological grade				
(I + II)	120	82 (68.3)	38 (31.7)	0.70
(III)	20	13 (65)	7 (35)	
Molecular subtypes				
Luminal A	51	34 (66.6)	17 (33.4)	0.093
Luminal B	53	36 (67.9)	17 (32.1)	
Her2neu Enr	18	16 (88.8)	2 (11.2)	
TNBC	18	9 (50)	9 (50)	

### FOXP3 Promoter Methylation and Its Association With Clinical Variables

**FIGURE 3:**
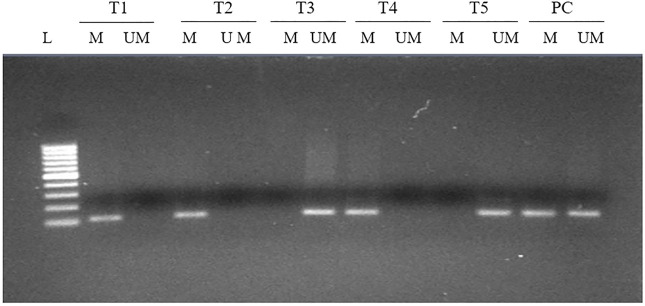
MS-PCR analysis of the promoter region of *FOXP3* in breast carcinoma. Promoter methylation was evaluated by two types of primers to amplify either unmethylated gDNA (UM) or methylated gDNA (M). L, 100-bp DNA ladder; T, tumor tissue; PC, positive control for completely methylated and unmethylated DNA.

Methylation at various CpG present in the upstream promoter area of *FOXP3* gene was observed in 73 cases (73/140, 52.14%) (Figure 3) and, once linked with clinical parameters, revealed a significant association with the Nottingham histological grades 1 and 2 type tumors of breast cancer patients (0.031). Though no significant associations were seen with other parameters included in the study (Table 3), we did note a significantly higher number of methylated cases in metastatic lymph node (59/73, 80.8%), estrogen receptor-positive (52/73, 71.2%), and menopausal female patients (51/73, 69.8%) (Table 3).

**TABLE 3:** Statistical association study of methylated *FOXP3* gene with clinicopathological parameters of patients having breast carcinoma.

Characteristics	Total cases (N)	Methylated	Unmethylated	*p-*value
Age				
<50	50	30 (60)	20 (40)	0.217
≥50	90	43 (47.7)	47 (52.3)	
Menopausal status				
Premenopausal	40	22 (55)	18 (45)	0.711
Postmenopausal	100	51 (51)	49 (49)	
Estrogen receptor status				
Negative	37	21 (56.7)	16 (43.3)	0.568
Positive	103	52 (50.4)	51 (49.6)	
Progesterone receptor status				
Negative	67	34 (50.7)	33 (49.3)	0.866
Positive	73	39 (53.4)	34 (46.6)	
Her2 neu status				
Negative	70	37 (52.8)	33 (47.2)	1.0
Positive	70	36 (51.4)	34 (48.6)	
Tumor size				
<5	64	35 (54.6)	29 (45.4)	0.613
≥5	76	38 (50)	38 (50)	
Lymph node status				
Positive	119	59 (49.5)	60 (50.5)	0.164
Negative	21	14 (66.6)	7 (33.4)	
TNM staging				
Stage (I + II)	45	25 (55.5)	20 (44.5)	0.50
Stage (III + IV)	95	48 (50.5)	47 (49.5)	
Histological grade				
(I + II)	120	58 (48.3)	62 (51.7)	0.031*
(III)	20	15 (75)	5 (25)	
Molecular subtypes				
Luminal A	51	29 (56.8)	22 (43.2)	0.136
Luminal B	53	24 (45.2)	29 (54.8)	
Her2neu Enr	18	13 (72.2)	5 (27.8)	
TNBC	18	7 (38.8)	11 (61.2)	

### Convincing Association of FOXP3 Protein Expression With FOXP3 Promoter Methylation in the Cases of Breast Carcinoma

The correlation study of methylated *FOXP3* gene and its respective protein expression displayed the significant link, in which out of 95 cases with protein loss, 70 cases possessed methylation at the promoter region (70/95, 73.68%) (Table 4), whereas 25 cases were completely unmethylated (25/95, 26.3%) (Table 4). To add more, in 67 unmethylated samples (67/140, 47.8%), noticeably 62.6% (42/67) cases showed the presence of protein. Therefore, a potential statistical relation was observed between the *FOXP3* protein expression and its promoter methylation (*p* = 0.0001) (Table A4).

**TABLE 4:** Correlation study between FOXP3 protein expression and its promoter methylation in patients with breast cancer.

*FOXP3* promoter	*FOXP3* protein expression		Total (%)	*p* value	OR value (95% CI)
—	Absent	Present	—	0.0001*	0.039 (0.011-0.13)
Methylated	70	3	73 (52.1)		
Unmethylated	25	42	67 (47.9)		
Total	95 (67.8)	45 (32.2)	140		

OR, odds ratio; CI, confidence interval; *p*-value, Fischer’s exact test

### Association of Methylated FOXP3 Gene Exhibiting Loss of Protein With Numerous Clinicopathological Variables

The methylated promoter region of *FOXP3* cases demonstrating either the absence or presence of protein exhibited a statistically significant relation with Her 2 neu receptor (*p* = 0.004) and metastatic lymph node tumors (*p* = 0.01) (Table 5). Moreover, 95.7% of methylated cases (67/70) with lymph node metastasis have protein loss. (Table 5). Additionally, the cases having protein loss exhibiting either a methylated or unmethylated *FOXP3* promoter region shows a convincing association with positive Her-2 receptor (*p* = 0.03) and tumors of grades 1 and 2 (*p* = 0.01) (Table 5). Furthermore, it is seen that there is a strong statistical relation between *FOXP3* protein loss and the promoter methylation with the various clinicopathological parameters (Table 6), where most of the features were associated in a highly significant manner (*p*-value < 0.05).

**TAble 5:** Significant association of promoter methylation and its protein expression in patients with methylated *FOXP3* promoter or *FOXP3* Protein expression loss with various clinicopathological features of breast carcinoma.

Clinical characteristics	Total (N)	Methylated *FOXP3*		*p*-value	Total (N)	*FOXP3* loss		*p-*value
		*FOXP3* absent	*FOXP3 p*resent			Methylated *FOXP3FOXP3*	Unmethylated	
Age								
Age < 50	30	29	1	1.0	36	29	7	0.3
Age ≥ 50	43	41	2	—	59	41	18	—
Menopausal status								
Premenopausal	22	22	0	0.54	27	22	5	0.3
Postmenopausal	51	48	3	—	68	48	20	—
ER status								
Negative	21	21	0	0.55	26	21	5	0.4
Positive	52	49	3	—	69	49	20	—
PR status								
Negative	34	33	1	1.0	46	33	10	0.6
Positive	39	37	2	—	49	37	15	—
HER2 status								
Negative	13	10	3	0.004*	19	10	9	0.03*
Positive	60	60	0	—	76	60	16	—
Tumor size								
<5	35	33	2	0.6	46	33	13	0.8
≥5	38	37	1	—	49	37	12	—
Lymph node status								
Positive	68	67	1	0.01*	90	67	23	0.6
Negative	5	3	2	—	5	3	2	—
Clinical stage								
Stage (1 + 2)	22	20	2	0.20	25	20	5	0.5
Stage (3 + 4)	51	50	1	—	70	50	20	—
Histological stage/grade								
Stage (1 + 2)	62	60	2	0.39	75	60	15	0.01*
Stage (3)	11	10	1	—	20	10	10	—
Molecular subtypes								
Lum A	29	27	2	—	34	27	7	—
Lum B	24	23	1	0.6	36	23	13	0.4
HER2 Neu	13	13	0	—	16	13	3	—
TNBC	7	7	0	—	9	7	2	—

**TABLE 6:** Association study between methylated *FOXP3* and FOXP3 protein expression in stratification with clinicopathological features.

Clinical characteristics	Total (N)	*FOXP3* methylation status	FOXP3 expression		*p-*value
			Absent	Present	
Age					
Age < 50	30	M	29	1	0.0001*
		U	7	13	
Age ≥ 50	43	M	41	2	0.0001*
		U	18	29	
Menopausal status					
Premenopausal	22	M	22	0	0.0001*
		U	5	13	
Postmenopausal	51	M	48	3	0.0001*
		U	20	29	
ER status					
Negative	21	M	21	0	0.0001*
		U	5	11	
Positive	52	M	49	3	0.0001*
		U	20	31	
PR status					
Negative	34	M	33	1	0.0001*
		U	10	23	
Positive	39	M	37	2	0.001*
		U	15	19	
HER2 status					
Negative	37	M	35	2	0.0001*
			9		
		U		24	
Positive	36	M	35	1	0.0001*
		U			
			16	18	
Tumor size					
<5	35	M	33	2	0.0001*
		U	13	16	
≥5	38	M	37	1	0.0001*
		U	12	26	
Lymph node status					
Positive	59	M	57	2	0.0001*
		U	23	37	
Negative	14	M	13	1	0.005
		U	2	5	
Clinical stage					
Stage (1 + 2)	22	M	20	2	0.07
		U	15	8	
Stage (3 + 4)	51	M	50	1	0.0001*
		U	10	34	
Histological stage/grade					
Stage (1 + 2)	62	M	60	2	0.0001*
		U	20	38	
Stage (3)	11	M	10	1	0.12
		U	5	4	
Molecular subtypes					
Lum A	29	M	27	2	0.0001*
		U	7	15	
Lum B	24	M	23	1	0.0001*
		U	13	16	
Her2 neu	13	M	13	0	0.06
		U	3	2	
TNBC	7	M	7	0	0.002
		U	2	9	

## Discussion

*FOXP3* expression is identified in tumors of the breast, prostate, lung, gastric, and thyroid (Liu et al., 2015; Yang et al., 2017; Ma et al., 2014; the Chu et al., 2015) suggesting its crucial role in the biology of cancer. The previous study demonstrated the inverse correlation between breast cancer angiogenesis and nuclear *FOXP3* expression. Adding to the same observation, the significant downregulation of *FOXP3* also resulted in the reduced survival in breast cancer (Li et al., 2018) *FOXP3* has been reported to modulate the expression of various genes involved in the process of carcinogenesis to exert its suppressing role in tumor development (Szylberg et al., 2016). At the same time, we cannot forget that significant studies have suggested the positive association between *FOXP3* expression and better survival in patients and the tumor-suppressive role of the *FOXP3* gene in breast cancer. Therefore, the present work investigated the *FOXP3* expression pattern and its correlation with various clinicopathological variables to strengthen its prognostic value and tumor-suppressive property. An earlier study demonstrated a quantitative method to assess the methylation status of *FOXP3* to understand the role of Treg cells in immunomodulation (Wieczorek et al., 2009). In our study transcription factor, *FOXP3* promoter methylation and expression were studied and analyzed in breast cancer patients of the northern region of India using methylation-specific PCR, real-time PCR, and immunohistochemistry to assess its role as a potential biomarker. The study correlated the findings with the clinicopathological variables (age, histological grade, ER status, HER2 status, *etc*.) of the procured cases.

In our study, at the mRNA level, nearly 63.5% (89/140) of cases were found to be downregulated (5.09 fold), and interestingly 86.5% (77/89) were linked with the histological grades I and II, suggesting the possible role of *FOXP3* in the early development of the disease. The study is supported by the previous studies on *FOXP3* expression at the transcript level (Zhang and Sun, 2010; Hinz et al., 2007). Furthermore, apart from breast cancer, one of the studies pointed out that the upregulation of *FOXP3* in gastric cancer cells put a brake on GC cell growth in both *in vivo* and *in vitro* studies (Hao et al., 2014), unraveling the crucial role of *FOXP3* expression in different carcinomas.

The protein expression profile exhibited low or no expression in nearly 69% (95/140) of breast cancer cases, followed by either moderate or high expression in 32% (45/140) of the cases. The expression was either cytoplasmic or nuclear, which was demonstrated in different types of cancer (Merlo et al., 2009; Karanikas et al., 2008; Zhang and Sun, 2010; Hinz et al., 2007; Ladoire et al., 2011; Tao et al., 2012). We did find a significant association between *FOXP3* protein expression and the TNM stage (*p*-value, 0.035). Interestingly almost 74% (95/140) of the cases of stage (III and IV) harbored protein loss. Because of the above mentioned condition, it has been observed earlier that, in the most aggressive cancer of epithelial tissues, *FOXP3* may help in the suppression of cancer as these aggressive cancer tissues harbored very low or no expression of *FOXP3* at the transcript and protein levels (Wang et al., 2009; Jiang and Ruan, 2014; Li et al., 2011).

The promoter methylation of *FOXP3* was observed in 52% of the cases (73/140) and significantly associated with Nottingham histological grades 1 and 2 (*p*-value 0.031). The finding depicts a strong association between promoter hyper-methylation and mRNA expression in deactivating or down-regulating the possible role of *FOXP3* in the suppression of breast cancer (Esteller, 2007; Li et al., 2007).

While analyzing an association between protein loss and hypermethylated promoter cases, we found a compelling association as out of 95 protein loss cases, 70 cases possessed methylation at the promoter region (70/95, 73.68%), whereas in a total of 67 unmethylated cases, 62.6% (42/67) exhibited the presence of protein. More intriguing results came out while analyzing methylation and protein loss with each other, as 95.7% methylated cases (67/70) with lymph node metastasis displayed protein loss. The findings are strongly supported by previous studies that have shown the association to be significantly lower in tumor grade and lymph node involvement in the breast tumor cells with positive *FOXP3* expression (Ladoire et al., 2012). Thus, the loss in *FOXP3* due to epigenetic change like methylation may serve as a potential biomarker in cases with lymph node metastasis.

In summary, our data provide some intriguing findings of *FOXP3* expression and its association with different clinicopathological parameters. However, the present study on a smaller sample size may weaken the statistical power. Therefore, further investigation on different sets of the population with a larger sample size is required to establish *FOXP3* as a potential cancer biomarker for diagnostic and prognostics purposes.

## Data Availability

All datasets generated for this study are included in the article/supplementary material.
